# Association Between the Cerebral Autoregulation Index (Pressure Reactivity), Patient’s Clinical Outcome, and Quality of ABP(t) and ICP(t) Signals for CA Monitoring

**DOI:** 10.3390/medicina56030143

**Published:** 2020-03-20

**Authors:** Basant K. Bajpai, Aidanas Preiksaitis, Saulius Vosylius, Saulius Rocka

**Affiliations:** 1 Health Telematics Science Institute, Kaunas University of Technology, LT-51423 Kaunas, Lithuania; 2 Centre of Neurosurgery, Clinic of Neurology and Neurosurgery, Faculty of Medicine, Vilnius University, LT08661 Vilnius, Lithuania; 3 Clinic of Anesthesiology and Intensive Care, Institute of Clinical Medicine, Faculty of Medicine, Vilnius University, LT08661 Vilnius, Lithuania

**Keywords:** arterial blood pressure, cerebral perfusion pressure, cerebrovascular autoregulation, intracranial pressure, pressure reactivity index, cerebral blood flow, receiver-operating characteristic, area under the curve, traumatic brain injury

## Abstract

*Background and Objectives*: The aim of this study was to explore the association between the cerebral autoregulation (CA) index, the pressure reactivity index (PRx), the patient’s clinical outcome, and the quality of arterial blood pressure (ABP(t)) and intracranial blood pressure (ICP(t)) signals by comparing two filtering methods to derive the PRx. *Materials and Methods*: Data from 60 traumatic brain injury (TBI) patients were collected. Moving averaging and FIR (Finite Impulse Response) filtering were performed on the ABP(t) and ICP(t) signals, and the PRx was estimated from both filtered datasets. Sensitivity, specificity, and receiver-operating characteristic (ROC) curves with the area under the curves (AUCs) were determined using patient outcomes as a reference. The outcome chosen for comparison among the two filtering methods were mortality and survival. *Results*: The FIR filtering approach, compared with clinical outcome, had a sensitivity of 70%, a specificity of 81%, and a level of significance *p* = 0.001 with an area under the curve (AUC) of 0.78. The moving average filtering method compared with the clinical outcome had a sensitivity of 58%, a specificity of 72%, and a level of significance *p* = 0.054, with an area under the curve (AUC) of 0.66. *Conclusions*: The FIR (optimal) filtering approach was found to be more sensitive for discriminating between two clinical outcomes, namely intact (survival) and impaired (death) cerebral autoregulation for TBI treatment decision making.

## 1. Introduction

Severe traumatic brain injury (TBI) is one of the primary causes of traumatic death worldwide. In Europe alone, 2.5 million people suffer TBI every year, with one million being admitted to the hospital. Approximately 30–35% of deaths result from such injuries, though it should be noted that some suffer from disabilities [1,2]. More attention and research are required to better understand and improve the management of severe TBI.

The main objective of clinical TBI studies is to improve the management of severe TBI, and the main factor that influences treatment outcomes is cerebral autoregulation (CA) [2,3]. Autoregulation has been previously described as a balancing act between vasoconstriction and vasodilation, as the resistance of the cerebrovascular bed accepts slow dynamic changes in cerebral perfusion pressure. CA impairment is most likely to influences the outcomes, and thus, it is essential to explore CA over time continuously [4,5].

Czosnyka et al. described the relationship between slow changes in mean arterial blood pressure (ABP) and intracranial pressure (ICP), which led to a better understanding of the relationship between cerebral perfusion pressure (CPP) and cerebral blood flow (CBF) by using the PRx [5,6]. This index is most commonly obtained by calculating the Pearson correlation between slow wave ABP and ICP [7,8,9]. With intact CA, slow increases in ABP cause vasoconstriction, which is followed by a decrease in ICP, resulting in a negative PRx; however, while CA is impaired, a rise in luminal ABP leads to passive cerebrovascular dilation and increases in cerebral blood volume and ICP. In such cases, the correlation coefficient (PRx) between ABP and ICP is positive [10,11].

The critical threshold for the pressure reactivity index has been suggested and recommended by various researchers (i.e., a PRx above 0.2 or 0.25 associated with impaired status and close to zero or a negative PRx associated with intact autoregulation) [12,13,14,15,16,17,18,19,20]. Among these PRx thresholds, PRx for survival (0.25) and that for favorable outcome (0.05) proposed by Sorrentino et al., [15] represent the reliable threshold for the estimation of survival and achieving a favorable outcome.

Moreover, a recent study by Akhondi-Asl et al. [21] found that PRx can only be evaluated when there are slow, but sufficient changes in ABP(t) and ICP(t) waves. The results revealed the sensitivity of the PRx calculation towards small slow wave changes in ABP(t) and ICP(t), and only slight PRx variance was observed [21], which indicated that if ABP(t) and ICP(t) waves changed with a filtering approach, the diagnostic value of the PRx may change.

The short period mean of ABP(t) and ICP(t) is needed to calculate the PRx [5,6,7,8,9,10,11,12,13,14,15,16,17,18,19]. The most common approach uses a moving average. It has been argued that another filtering approach (optimal filters) for short period means of ABP(t) and ICP(t) is better than using the moving average to estimate the mean pressure [22]. However, the impact of an optimal filtering approach on PRx has not been evaluated. Therefore, we analyzed ABP(t) and ICP(t) signals with an optimal filter and compared the results with the quality of the signal using Czosnyka et al.’s moving average. The sensitivity and specificity of the PRx were evaluated, with the quality of the signal based on the clinical outcomes. The Glasgow outcome scale (GOS) score after hospital discharge (GOSHD) was used as the clinical reference (the mortality and survival). GOS outcomes were taken as the reference for the dichotomous outcome for sensitivity, specificity, and ROC curve estimation. The patient’s outcome was defined using the following: 1 (death), 2 (persistent vegetative state), 3 (severe disability), 4 (moderate disability), and 5 (low disability) [23].

A single CA impairment event lasts for approximately 5 min and is strongly associated with postoperative cognitive dysfunction and cognition deficits [24], which means a CA monitor must have a time resolution of one minute or less, corresponding to the duration of the output reaction of a filter to an input step function for ABP(t) or ICP(t)). Such a CA monitor would show the start of severe CA impairment with a delay of about 1 min. In this case, an optimal filter with a 1 min time window would be applicable.

## 2. Materials and Methods

The methods were carried out in accordance with the approved guidelines. This study was approved by the Vilnius Regional Ethics Committee of Biomedical Studies (Vilnius, Lithuania), Approval No. 158200-15-801-323, which was conducted from 2015–2019 in the intensive care unit (ICU) at the Republican Vilnius University Hospital (Lithuania), with prior written informed consent being obtained from all patients. Clinical data from 60 TBI patients were collected using an ICP intraparenchymal transducer (Codman microsensor ICP transducer). The arterial blood pressure was invasively measured using an ABP monitor (Datex-Ohmeda GmbH, Duisburg, Germany). Further data recording and processing were accomplished using ICM+ software (Cambridge Enterprises Ltd., Cambridge, UK), where the real-time PRx values were collected. All patient data were used post hoc to obtain additional information and analyze real-time data over time.

### 2.1. Post-Hoc Analysis

A sampling frequency of 50 Hz was used to generate raw ABP and ICP data. The recorded data were reduced to a 1 Hz sampling frequency and filtered with the following two filters:

A. Moving average data filter: The mean values of every ABP(t) and ICP(t) signal were calculated for 5 s. The calculation was performing using the moving average, and PRx1 was calculated as a linear Pearson correlation for a 5 min time window between 60 consecutive values of mean arterial pressure (MAP) and ICP. The data points included in the analysis were between 50 and 120 mmHg for ABP and ICP values that were greater than zero. The average PRx1 was used for the sensitivity estimation of the ABP(t) and ICP(t) signal quality. This was considered the first method employed for sensitivity estimation.

B. Optimal FIR data filter: In FIR (Parks–McClellan) optimal filtering, the mean ABP and ICP were obtained by continuous filtering with a 1 min period of optimal filtering, and PRx2 was calculated as the linear Pearson correlation coefficient with 5 min segments of ICP and ABP signals. The FIR filter was designed for a 1 Hz sampling frequency with a passband ripple of 0.1 dB. The Parks–McClellan algorithm for designing FIR filters [22,25] was used to obtain mean pressure, which had never been used for PRx estimation. There are certain advantages to using an FIR filter, such as not increasing computational costs, less sensitivity to artifacts, shorter delays, and higher sensitivity to acute events. With FIR filters, it is easy to enforce the linear phase constraint if it is stable, and the duration of disruptions is limited to the impulse response duration, which is the filter length [22,25].

### 2.2. Statistical Analysis

Statistical analysis was performed using the software package SPSS (IBM Inc., New York, NY, USA) Version 20. Patients included in the study were grouped into those who survived and those who did not. For each group, average values for PRx1 (from Czosnyka et al.’s moving average filtering) and PRx2 (from optimal FIR filtering) were created (Table 1). An independent *t*-test was used to compare the categorical outcomes (i.e., survivals/non-survivals based on GOS outcomes) and the continuous outcome.

Two additional Tables (Table 2 and Table 3) were created for sensitivity and specificity calculations with true positive (TP), true negative (TN), false positive (FP), and false negative (FN) from the PRx outcome of both filtering methods. The GOS scale (i.e., a GOS value of 1 corresponding to death and a GOS value greater than 1 indicative of survival [23]) was considered as the reference clinical outcome. The GOS outcomes were taken as the dichotomous outcomes for true positive (TP), true negative (TN), false positive (FP), and false negative (FN) estimation, hence sensitivity and specificity.

Two ROC (receiver operating characteristic) curves were constructed in one window for 60 patients for each filtering method to compare the effectiveness of the two filtering methods. The diagnostic accuracy of the ROC curve was represented by the area under the curve (AUC). AUC values closer to 1 indicated that the screening measures were reliable [26,27,28,29,30].

## 3. Results

**Figure 1:**
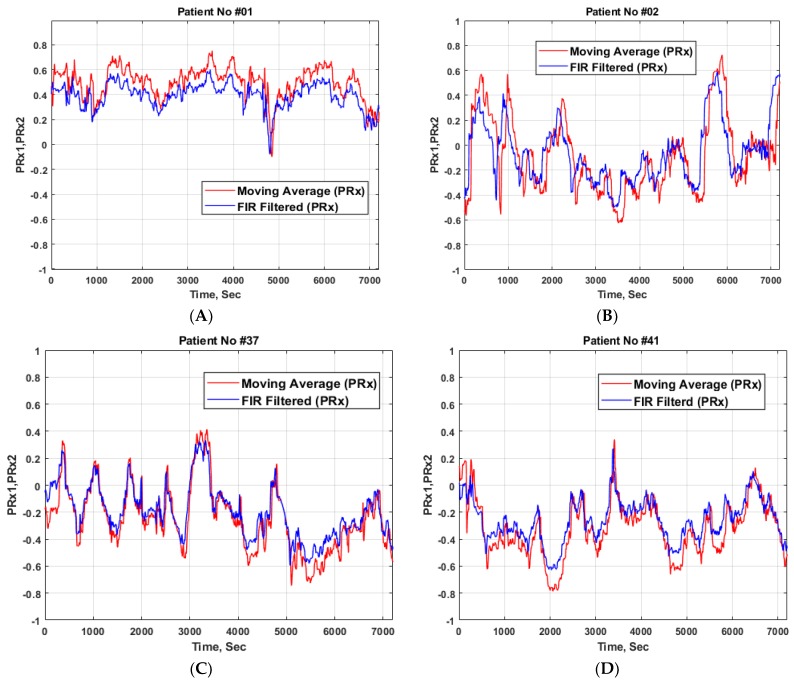
Example of a two hour period of PRx1 (moving average filtering) and PRx2 (FIR filtered data) comparison in patients with impaired cerebral autoregulation (CA) (**A**), intermediate CA (**B**,**C**), and intact CA (**D**).

Both PRx values from Czosnyka et al.’s moving average filtering (PRx1) and optimal FIR filtering (PRx2) were estimated for comparative purposes for all 60 TBI patients. Demographic patient data are shown in Table 1, where among 60 patients, 34 male and nine female patients were survivors, while 13 male and four female patients did not survive (fatal). Moreover, the comparative pressure reactivity index of two hours of PRx1 (Czosnyka et al.’s moving average filtering) and PRx2 (FIR filtering) are presented in Figure 1A–D, including patients with impaired CA (A), intermediate CA (B, C), and intact CA (D).

### 3.1. Sensitivity and Specificity

As shown in Table 2, patients were grouped according to PRx outcomes, where the PRx2 from the FIR filtering approach included 12 patients with true positives, 35 with true negatives, 8 with false positives, and 5 with false negatives, which reflected a sensitivity of 70% and a specificity of 81% (by the FIR filtering approach).

Sensitivity = True Positive/(True positive + False negative) = 12/17 = 70%

Specificity = True Negative/(False positive + True negative) = 35/43 = 81%

As shown in Table 3, patients were grouped according to PRx outcomes, where PRx1 from Czosnyka et al.’s moving average filtering approach included 10 patients with true positives, 31 with true negatives, 13 with false positives, and 7 with false negatives, which reflected 58% sensitivity and 72% specificity by the FIR filtering approach.

Sensitivity = True Positive/(True positive + False negative) = 10/17 = 58%

Specificity = True Negative/(False positive + True negative) = 35/43 = 72%

### 3.2. Receiver Operating Characteristic Curve

The ROC curve was constructed from 60 patients’ PRx data using both filtering approaches, true positives (PRx associated with impaired autoregulation), and true negatives (PRx associated with intact autoregulation), where true positives and true negatives were plotted against each other for the ROC curve from Table 2 and Table 3.

**Figure 2:**
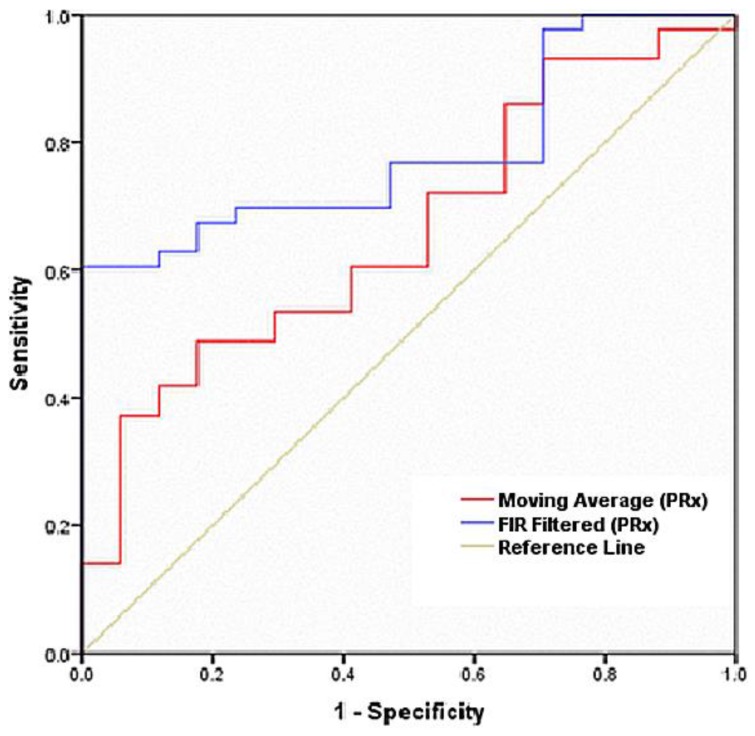
ROC curves for both methods’ PRx values, moving average filtering, and FIR optimal filtering. FIR filtered (PRx) shows better diagnostic accuracy with a larger area under the curve (AUC) compared to the moving average (PRx).

For the moving average of PRx1, the ROC curve (red) had an area under the curve (AUC) of 0.661 with a sensitivity of 58%, a specificity of 72%, and a significance level of 0.054. The area under the ROC curve had a standard error of 0.075 and a 95% confidence interval of 0.515–0.807, as shown in Figure 2 and Table 3 (ROC curve from both methods’ PRx values, moving average filtering, and FIR optimal filtering). For the FIR filtered PRx2, the ROC curve (blue) had an area under the curve (AUC) of 0.785, with a sensitivity of 70%, a specificity of 81%, and a significance level of 0.001. The area under the ROC curve had a standard error of 0.058 and a 95% confidence interval of 0.671–0.900, as shown in Figure 2 and Table 4 (ROC curve from both method’s PRx values, moving average filtering, and FIR optimal filtering).

### 3.3. Independent t-test

Independent *t*-tests between categorical (survival and non-survivals based on GOS outcome) and the continuous PRx from moving average PRx showed a significance level (Significance. (two tailed)) of 0.040, with a 95% confidence interval of 0.01428–0.57685 and a standard error of 0.13921. The FIR filtered data PRx showed a significance level (Sig. (two tailed)) of 0.001 with a 95% confidence interval of 0.20568–0.70401 and a standard error of 0.12151. This *t*-test indicated that FIR filtering had better significance and a lower standard error than moving average filtering, as shown in Table 5.

## 4. Discussion

In this study, the association between the CA index (PRx) associated with a patient’s clinical outcomes (from GOS scale) and the quality of the signal to estimate the CA index (PRx) was estimated. Czosnyka et al. described the relationship between slow changes in mean ABP and ICP, which led to the development of the relationship between CPP and CBF by using the PRx [5,6]. This index was obtained by calculating the Pearson correlation between slow wave ABP and ICP [7,8,9]. With intact CA, slow increases in ABP caused vasoconstriction, which was followed by a decrease in ICP, resulting in a negative PRx. However, while the CA was impaired, a rise in luminal ABP led to passive cerebrovascular dilation and increases in the cerebral blood volume and ICP. In such cases, the correlation coefficient (PRx) between ABP and ICP was positive [10,11].

The short period means of ABP(t) and ICP(t) were essential for estimating the PRx [5,6,7,8,9,10,11,12,13,14,15,16,17,18,19]. According to prior studies, ICP signals are often polluted by a significant number of artifacts. Artifacts contaminate an average of 5% of data points in the collected ICP signals, and in some cases, more than 20% of signals can be polluted [31]. The most common approach employed to obtain a short period means the moving average. It has been argued that optimal FIR filters for the short period means of ABP(t) and ICP(t) are better than the moving average to estimate the mean pressure [22]. Akhondi-Asl et al. stated that the PRx could only be evaluated when there are slow, but sufficient changes in ICP and ABP waves. This shows the sensitivity of the PRx calculation to slow wave changes, and only slight PRx variance can be seen with small slow wave changes [21]. In other words, if there are higher or lower wave changes in ABP(t) and ICP(t), the diagnostic PRx value would be different.

Hence, we used the FIR optimal filters for the PRx estimation, which is an essential index for CA monitoring. In our study, we evaluated the sensitivity and specificity of the PRx, basing the quality of the signal on the clinical outcomes. The Glasgow outcome scale (GOS) score after hospital discharge (GOSHD) was used as a clinical reference (mortality and survival). GOS outcomes were taken as the reference for the dichotomous outcome for sensitivity, specificity, and ROC curve estimation. The patient’s outcome was defined using the following five point scale: 1 (death), 2 (persistent vegetative state), 3 (severe disability), 4 (moderate disability), and 5 (low disability) [23].

From the ROC curve, the FIR filter method revealed a sensitivity of 70%, a specificity of 81%, an area under the ROC curve of 0.78, and a significance level of *p* = 0.001, with a standard error of 0.058. These results were better than those found with the moving average method, which had a sensitivity of 58% and a specificity of 72%. The area under the ROC curve was 0.661, and the significance level was *p* = 0.054. The area under the ROC curve revealed a standard error of 0.075. An AUC closer to 1.0 is ideal for discriminative values between healthy and sick patients [25,26,27,28,29]. The ROC curve reflected that the FIR filter approach was better than the moving average method for ABP and ICP signal filtering. The *t*-test reflected that FIR filtering results with a better significance level (0.001) and lower standard error (0.12151) than the moving average (significance level 0.040, with a standard error of 0.13921), as shown in Table 4.

FIR filtering resulted in better time resolution because of the optimal filter with a 1 min time window. A single CA impairment event is approximately 5 min long and is strongly associated with postoperative cognitive dysfunction and deficit of cognition [23]. That means that CA monitoring must have a time resolution of 1 min or less, which is the duration of the output reaction of the filter to an input step function of ABP(t) or ICP(t)). Such CA monitoring would show the start of severe CA impairment with a delay of about 1 min.

## 5. Conclusions

The association between the sensitivity of the PRx, brain-injured patient’s clinical outcome, and the quality of ABP(t) and ICP(t) indicated that the FIR (optimal) filtering approach was more sensitive for discriminating between two clinical outcomes, namely intact (survival) and impaired (death) cerebral autoregulation for TBI treatment decision making. ABP(t) and ICP(t) signal analysis in TBI patients is particularly essential to minimize the risk of uncertain diagnostic values (intact or impaired) of the PRx.

### Limitations of the Study

This study was conducted on a small population of patients (60 patients). A validation study with a much larger population is necessary. Furthermore, there is no gold standard for the cerebral autoregulation monitoring method. Another essential factor for producing more concrete results is the comparison of PRx outcomes with various filtering approaches and various methods.

## Figures and Tables

**Table 1:** Demographic characteristics, clinical findings, and averaged values of data from monitoring TBI patients.

	Survival	Fatal	Total	*p*-value
Number of patients	43	17	60	-
Sex (male/female)	34/9	13/4	47/13	-
Age, mean (SD), years	36.84 (16.09)	43.25 (11.64)	38.52 (15.35)	0.028
GCS, median	6 (4–7)	5 (4–6)	5.50 (5–6)	0.015
Average PRx1 (moving average filtered), mean (SD)	0.08 (0.31)	0.21 (0.34)	0.11 (0.31)	0.007
Average PRx2 (optimal filtered), mean (SD)	−0.01(0.36)	0.26 (0.22)	0.01(0.37)	0.001

GCS, Glasgow coma scale; PRx, pressure reactivity index; SD, standard deviation; TBI, traumatic brain injury.

**Table 2:** 2 × 2 matrix (FIR filtered data PRx2).

Outcome	Impaired Autoregulation/Non-survivors (Fatal)	Intact Autoregulation/Survivors
**Positive test** (GOS * 1)	(True positive) 12 (M-9, F-3)	(False positive) 8 (M-6, F-2)
**Negative test** (GOS *>1)	(False negative) 5 (M-5, F-0)	(True negative) 35 (M-27, F-8)

* GOS, Glasgow scale estimation of clinical outcome; M, male; F, female.

**Table 3:** The 2 × 2 matrix (moving average PRx1).

Outcome	Impaired Autoregulation/ Non-survivors (Fatal)	Intact autoregulation/ Survivors
**Positive Test** (GOS * 1)	(True positive) 10 (M-8, F-2)	(False positive) 13 (M-10, F-3)
**Negative test** (GOS *>1)	(False negative) 7 (M-5, F-2)	(True negative) 31 (M-25, F-6)

* GOS, Glasgow scale estimation of clinical outcome; M, male; F, female.

**Table 4:** Results for the area under the ROC curve (AUC) obtained for the moving average and FIR filter criterion values and coordinates of the ROC curve(show).

Test Result Variable(s)	Area	Std. Error *	Asymptotic Significance. **	Asymptotic 95% Confidence Interval	
				Lower Bound	Upper Bound
Moving Average (PRx)	0.661	0.075	0.054	0.515	0.807
FIR Filtered (PRx)	0.785	0.058	0.001	0.671	0.900

* Under the nonparametric assumption. ** Null hypothesis: true area = 0.5. FIR filtered (PRx2) shows better diagnostic accuracy with a higher area under the curve (AUC) compared to the moving average (PRx).

**Table 5:** Results for the independent *t*-test obtained from the moving average and FIR filter PRx criterion values and coordinates of the ROC curve (show).

Test Variable (s)	t	Sig.	df	Sig. (2-tailed)	Mean Difference	Std. Error Difference	95% Confidence Interval	
							Lower	Upper
Moving average PRx	2.123	0.054	40.319	0.040	0.29557	0.13921	0.01428	0.57685
FIR PRx	3.743	0.000	27.370	0.001	0.45484	0.12151	0.20568	0.70401
